# Supplementary material for: Parasite diversity and ecology in a model species, the guppy (Poecilia reticulata) in Trinidad
Source: R Soc Open Sci. 2020 Jan 22;7(1):191112. doi: 10.1098/rsos.191112 (PMC7029902; doi:10.1098/rsos.191112)
Supplement: Supplementary Information 1 [file rsos191112supp1.docx]

**Supplementary Information


Observations**

While housed in aquaria and awaiting necropsy, fish collected from Lopinot showed higher rates of mortality than those from other localities. The average total parasite burden of Lopinot fish (77 parasites/host) was more than double that of any other population. Lopinot fish were distinguished by higher infections with *G. bullatarudis* (Lopinot mean abundance 45.7, non-Lopinot mean abundance 2.3), Digenea sp. 1 (Lopinot mean abundance 8.8, non-Lopinot mean abundance 0.6) and *Ascocotyle* sp. (Lopinot mean abundance 6.5, non-Lopinot mean abundance 0.5).

While *G. bullatarudis* was the most common and abundant parasite observed, in most respects metacercariae of digenetic trematodes dominated parasites communities of Trinidadian guppies (Figure 1). Metacercariae also account for over one third of species of parasites ever recorded in guppies (28 of all 78 species, 11 of 31 natural infections, Table S8). The dominance of these trophically transmitted larval stages has implications for guppy evolution and ecology because parasite life cycles converge on evolutionarily stable trophic and ecological links (Marcogliese and Cone 1997; Poulin 2011). In fish and other aquatic hosts, the diversity and abundance of larval parasites is correlated with the diversity of their predators, including non-fish predators (Hechinger and Lafferty 2005; Chen et al. 2008; Anderson and Sukhdeo 2011; Locke et al. 2014). The three species of metacercariae we identified all mature in avian hosts, and this is likely true of the majority of the unidentified metacercariae, as nearly all trematodes recorded in guppies mature in birds (Table S8).

Among the non-gyrodactylid parasites encountered, several are likely to exert selective pressure on guppy populations due to pathogenesis. For example, fish from Lopinot had high mortality and were characterized by high total parasite burdens and heavy infections of *G. bullatarudis* and metacercariae of two digeneans (Figure 1). One of the latter occurred in the gills, where metacercariae can cause lesions (Mitchell et al. 2005). The other, *Ascocotyle* sp., was often present in high numbers (>10), causing the bulbus anteriosus of the heart to swell up to three times its normal size (see also Sogandares-Bernal and Lumsden 1964). In some guppies with visible deformities (scoliosis), we observed an unknown species of *Myxobolus* infecting the spine. Other species of *Myxobolus*, such as *M. cerebralis*, the causative agent of whirling disease, can also cause scoliosis and reduce swimming capacity in salmonids (Fetherman et al. 2011). Similar spinal deformities have been observed in guppies in other collections from the North Slope drainage and in previous years at other sites (pers. ob.). We also observed infrequent infections with *C. cotti*, which even in low numbers can reduce breeding success in guppies (Mcminn 1990). More generally, larval parasites such as metacercariae, which were highly abundant across the study system, are often pathogenic (Combes and Nassi 1977; Brassard et al. 1982; Ewald 1995; Levy et al. 2002). In six populations of *Poecila mexicana* in which metacercariae were dominant parasites, mean infracommunity abundance and diversity were strongly associated with the diversity of host immune genes (Tobler et al. 2014). In short, our data suggest parasites other than gyrodactylids appear likely to affect fish health in Trinidad, and should be considered in future studies of parasite-mediated selection and the evolutionary ecology of guppies.

**Results**

**
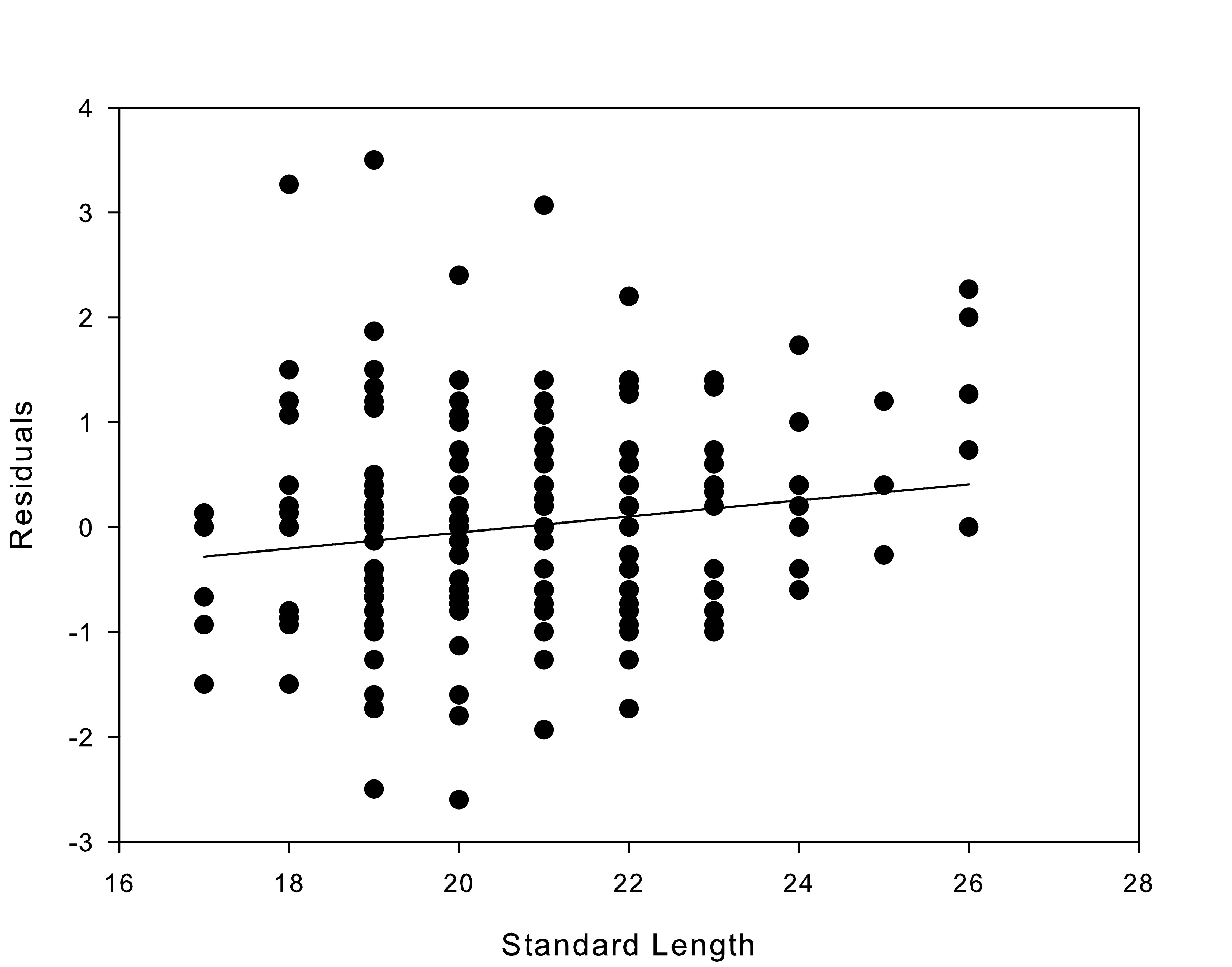
**

**Figure S1.** The relationship between standard length and the total number of parasites in an individual fish. On the y-axis are the residuals of a general linear model (GLM) of the number of parasite species (species richness) infecting an individual as response variable, with drainage nested within population as factors. These residuals are regressed against fish standard length (shown on the X-axis). Larger males show on average a higher parasite species richness (regression: F_1,257_=8.00, p=0.005, r^2^ adj=2.6%).

**Figure S2.** The relationship between the number of digenean species on a single host and the number of snail species in the population for the guppies of the Caroni Drainage, and the Northern Slope. Guppies in populations with a higher snail biodiversity are infected with significantly more species of digeneans across all populations (Pearson correlation: r=0.563, p<0.001), and separately in the North Slope (Pearson correlation: r=0.313, p<0.001), as well as in the Caroni Drainage (Pearson correlation: r=0.421, p<0.001).

**Figure S3.** Comparison of Jaccard’s dissimilarity shows that population are in general more differentiated by parasite community than they are by piscine predator community composition Kruskal-Wallis Test: H=17.62, df=7, p=0.013.

|  | **North Slope populations** | **Caroni populations** | **Binomial p** |
| --- | --- | --- | --- |
| *Ascocotyle* sp. | 3 | 5 | 0.157 |
| *Posthodiplostomum* sp. | 0 | 4 | **0.005** |
| *Pygidiopsis* sp. | 2 | 4 | 0.157 |
| *Saccocoelioides* sp. | 1 | 7 | **<0.001** |
| *Digenea* sp. 1 | 2 | 6 | **0.008** |
| *Digenea* sp. 2 | 0 | 7 | **<0.001** |
| *Digenea* sp. 3 | 1 | 0 | 1 |
| *Digenea* sp. 4 | 1 | 0 | 1 |
| *Digenea* sp. 5 | 2 | 0 | 1 |
| *Digenea* sp. 6 | 1 | 0 | 1 |
| *Digenea* sp. 7 | 0 | 2 | 0.104 |
| *Digenea* sp. 8 | 1 | 0 | 1 |
| *Digenea* sp. 9 | 1 | 0 | 1 |
| *Gyrodactylus bullatarudis* | 4 | 8 | **0.001** |
| *Gyrodactylus turnbulli* | 1 | 7 | **<0.001** |
| *Urocleidoides reticulatus* | 1 | 1 | 0.736 |
| *Camallanus* sp. | 0 | 2 | 0.104 |
| *Ambiphrya* sp. | 1 | 0 | 1 |
| *Myxobolus nuevoleonensis* | 0 | 1 | 0.346 |
| Fungi | 0 | 1 | 0.346 |
| *Trichodina* sp. | 0 | 5 | **<0.001** |

**Table S3**. A binomial test shows that seven species of parasites are significantly more common among populations of the Caroni Drainage than in populations of the North Slope

|  | Ascocotyle | Posthodi | Pygidiop | Saccocoe | Digenean1 | Digenean2 | G.bullatarudis | G.turnbulli |
| --- | --- | --- | --- | --- | --- | --- | --- | --- |
| Posthodi | **0.383** | * | * | * | * | * | * | * |
| p | **0.000** | * | * | * | * | * | * | * |
| Pygidiop | -0.049 | -0.094 | * | * | * | * | * | * |
| p | 0.573 | 0.280 | * | * | * | * | * | * |
| Saccocoe | 0.073 | -0.106 | -0.083 | * | * | * | * | * |
| p | 0.401 | 0.220 | 0.337 | * | * | * | * | * |
| Digenean1 | **0.495** | -0.046 | -0.050 | **0.340** | * | * | * | * |
| p | **0.000** | 0.599 | 0.565 | **0.000** | * | * | * | * |
| Digenean2 | **0.445** | 0.029 | -0.087 | 0.197 | **0.461** | * | * | * |
| p | **0.000** | 0.739 | 0.316 | 0.022 | **0.000** | * | * | * |
| G. bullarudis | 0.255 | -0.065 | -0.077 | 0.134 | **0.554** | **0.436** | * | * |
| P | 0.003 | 0.451 | 0.375 | 0.121 | **0.000** | **0.000** | * | * |
| G. turnbulli | -0.033 | -0.092 | 0.153 | -0.014 | 0.078 | 0.046 | 0.027 | * |
| p | 0.708 | 0.287 | 0.077 | 0.876 | 0.368 | 0.596 | 0.752 | * |
| Trichodina | **0.349** | -0.102 | 0.018 | 0.150 | **0.502** | **0.543** | **0.600** | 0.165 |
| p | **0.000** | 0.238 | 0.839 | 0.083 | **0.000** | **0.000** | **0.000** | 0.056 |

**Table S4a.** Pearson correlation (and p-value) between the number of parasites on individual guppies in the Caroni Drainage sites. Only parasite species are included that were recorded on 15 or more guppies.

|  | Ascocotyle | Posthodi | Pygidiop | Saccocoe | Digenean1 | Digenean2 | G.bullatarudis | G.turnbulli |
| --- | --- | --- | --- | --- | --- | --- | --- | --- |
| Posthodi | * |  | * | * | * |  | * | * |
| p | * |  | * | * | * |  | * | * |
| Pygidiop | -0.042 |  | * | * | * |  | * | * |
| p | 0.626 |  | * | * | * |  | * | * |
| Saccocoe | 0.051 |  | -0.034 | * | * |  | * | * |
| p | 0.558 |  | 0.693 | * | * |  | * | * |
| Digenean1 | -0.049 |  | 0.081 | -0.040 | * |  | * | * |
| p | 0.569 |  | 0.349 | 0.645 | * |  | * | * |
| Digenean2 | * |  | * | * | * |  | * | * |
| p | * |  | * | * | * |  | * | * |
| G. bullarudis | -0.060 |  | -0.018 | -0.049 | -0.070 |  | * | * |
| P | 0.489 |  | 0.838 | 0.576 | 0.418 |  | * | * |
| G. turnbulli | -0.016 |  | **0.221** | -0.013 | 0.068 |  | 0.111 | * |
| p | 0.853 |  | **0.010** | 0.881 | 0.432 |  | 0.198 | * |
| Trichodina | -0.016 |  | -0.020 | -0.013 | -0.023 |  | -0.028 | -0.007 |
| p | 0.853 |  | 0.821 | 0.881 | 0.792 |  | 0.748 | 0.932 |

**Table S4b**. Pearson correlation (and p-value) between the number of parasites on individual guppies in the Northern Slope sites.

| **Guanapo** | Ascocotyle | Posthodi | Pygidiop | Saccocoe | Digenean1 | Digenean2 | G.bullatarudis | G.turnbulli |
| --- | --- | --- | --- | --- | --- | --- | --- | --- |
| Posthodi | 0.481 | * | * | * | * | * | * | * |
| p | 0.070 | * | * | * | * | * | * | * |
| Pygidiop | **0.741** | 0.557 | * | * | * | * | * | * |
| p | **0.002** | 0.031 | * | * | * | * | * | * |
| Saccocoe | 0.018 | 0.323 | 0.054 | * | * | * | * | * |
| p | 0.949 | 0.241 | 0.850 | * | * | * | * | * |
| Digenean1 | **0.947** | 0.392 | 0.589 | -0.033 | * | * | * | * |
| p | **0.000** | 0.148 | 0.021 | 0.908 | * | * | * | * |
| Digenean2 | **0.803** | 0.343 | 0.370 | 0.045 | **0.822** | * | * | * |
| p | **0.000** | 0.211 | 0.175 | 0.873 | **0.000** | * | * | * |
| G. bullarudis | -0.224 | 0.222 | -0.004 | 0.275 | -0.342 | -0.276 | * | * |
| P | 0.422 | 0.427 | 0.989 | 0.322 | 0.212 | 0.319 | * | * |
| G. turnbulli | * | * | * | * | * | * | * | * |
| p | * | * | * | * | * | * | * | * |
| Trichodina | -0.125 | 0.034 | -0.140 | -0.128 | -0.154 | -0.020 | -0.184 | * |
| p | 0.658 | 0.903 | 0.618 | 0.648 | 0.583 | 0.944 | 0.513 | * |
|  |  |  |  |  |  |  |  |  |

**Table S5a**. Pearson correlation (and p-value) between the number of parasites on individual guppies in the Guanapo River in the Caroni Drainage.

| **Lower Aripo** | Ascocotyle | Posthodi | Pygidiop | Saccocoe | Digenean1 | Digenean2 | G.bullatarudis | G.turnbulli |
| --- | --- | --- | --- | --- | --- | --- | --- | --- |
| Posthodi | * | * | * | * | * | * | * | * |
| p | * | * | * | * | * | * | * | * |
| Pygidiop | * | * | * | * | * | * | * | * |
| p | * | * | * | * | * | * | * | * |
| Saccocoe | * | * | 0.159 | * | * | * | * | * |
| p | * | * | 0.571 | * | * | * | * | * |
| Digenean1 | * | * | 0.082 | -0.073 | * | * | * | * |
| p | * | * | 0.772 | 0.796 | * | * | * | * |
| Digenean2 | * | * | -0.220 | -0.167 | 0.252 | * | * | * |
| p | * | * | 0.431 | 0.553 | 0.366 | * | * | * |
| G. bullarudis | * | * | -0.098 | -0.430 | **0.552** | 0.308 | * | * |
| P | * | * | 0.728 | 0.109 | **0.033** | 0.264 | * | * |
| G. turnbulli | * | * | 0.048 | -0.427 | **0.635** | 0.228 | **0.867** | * |
| p | * | * | 0.866 | 0.113 | **0.011** | 0.414 | **0.000** | * |
| Trichodina | * | * | 0.072 | 0.308 | 0.207 | 0.039 | 0.287 | 0.386 |
| p | * | * | 0.798 | 0.264 | 0.459 | 0.891 | 0.300 | 0.155 |

**Table S5b**. Pearson correlation (and p-value) between the number of parasites on individual guppies in the Lower Aripo River in the Caroni Drainage.

| **Santa Cruz** | Ascocotyle | Posthodi | Pygidiop | Saccocoe | Digenean1 | Digenean2 | G.bullatarudis | G.turnbulli |
| --- | --- | --- | --- | --- | --- | --- | --- | --- |
| Posthodi | **0.813** | * | * | * | * | * | * | * |
| p | **0.004** | * | * | * | * | * | * | * |
| Pygidiop | -0.340 | -0.349 | * | * | * | * | * | * |
| p | 0.337 | 0.323 | * | * | * | * | * | * |
| Saccocoe | 0.027 | 0.445 | -0.554 | * | * | * | * | * |
| p | 0.942 | 0.198 | 0.097 | * | * | * | * | * |
| Digenean1 | -0.205 | 0.078 | 0.380 | 0.305 | * | * | * | * |
| p | 0.569 | 0.830 | 0.279 | 0.391 | * | * | * | * |
| Digenean2 | -0.134 | -0.328 | -0.325 | -0.163 | -0.172 | * | * | * |
| p | 0.712 | 0.355 | 0.360 | 0.652 | 0.635 | * | * | * |
| G. bullarudis | -0.250 | 0.136 | 0.294 | 0.519 | **0.922** | -0.222 | * | * |
| P | 0.486 | 0.709 | 0.409 | 0.124 | **0.000** | 0.538 | * | * |
| G. turnbulli | -0.292 | 0.125 | 0.223 | 0.620 | **0.864** | -0.199 | **0.973** | * |
| p | 0.414 | 0.730 | 0.537 | 0.056 | **0.001** | 0.582 | **0.000** | * |
| Trichodina | -0.208 | -0.356 | -0.280 | -0.128 | -0.135 | **0.960** | -0.215 | -0.156 |
| p | 0.565 | 0.312 | 0.433 | 0.724 | 0.710 | **0.000** | 0.552 | 0.667 |

**Table S5c**. Pearson correlation (and p-value) between the number of parasites on individual guppies in the Santa Cruz in the Caroni Drainage

**Table S6**. Presence/absence data of predatory piscine species at each location

|  | *Hoplias malabaricus* | *Synbranchus marmoratus* | *Anablepsoides hartii* | *Crenicichla  alta* | *Gobiomorus dormitor* | *Eleotris pisonis* |
| --- | --- | --- | --- | --- | --- | --- |
| Las Cuevas | 0 | 0 | 1 | 0 | 0 | 1 |
| Yarra 2 | 0 | 0 | 0 | 0 | 1 | 1 |
| Marianne 16 | 0 | 0 | 1 | 0 | 0 | 0 |
| Marianne3 | 0 | 0 | 1 | 0 | 0 | 0 |
| Petite Marianne | 0 | 0 | 1 | 0 | 0 | 0 |
| Madamas | 0 | 0 | 1 | 0 | 0 | 0 |
| Paria7 | 0 | 0 | 1 | 0 | 0 | 0 |
| San Souci | 0 | 0 | 0 | 0 | 1 | 1 |
| Mission | 0 | 0 | 0 | 0 | 1 | 1 |
| El Cedro 2 | 1 | 1 | 1 | 0 | 0 | 0 |
| Guanapo Quarry | 1 | 1 | 1 | 1 | 0 | 0 |
| Lopinot | 0 | 1 | 1 | 0 | 0 | 0 |
| Lower Arima | 1 | 1 | 1 | 1 | 0 | 0 |
| Upper Arima | 0 | 0 | 1 | 0 | 0 | 0 |
| Santa Cruz | 0 | 1 | 1 | 0 | 0 | 0 |
| Lower Aripo | 1 | 1 | 1 | 0 | 0 | 0 |
| Upper Naranjo | 0 | 0 | 1 | 0 | 0 | 0 |
| Blue Basin | 0 | 0 | 1 | 0 | 0 | 0 |

**Table S7**. Presence/absence data of snail species at each location

|  | *Melanoides*  *tuberculata* | *Gundlachia*  *sp.* | *Physella*  *acuta* | *Marisa*  *cornuarietis* | *Neritina*  *virginea* | *Pomacea*  *glauca* | *Tarebia*  *granifera* | *Pseudosuccinea*  *columella* | *Pyrgophorus*  *parvulus* | *Biomphalaria*  *sp.* |
| --- | --- | --- | --- | --- | --- | --- | --- | --- | --- | --- |
| Las Cuevas | 0 | 0 | 0 | 0 | 0 | 0 | 1 | 0 | 0 | 0 |
| Yarra 2 | 0 | 0 | 0 | 1 | 0 | 0 | 1 | 0 | 0 | 0 |
| Marianne 16 | 0 | 0 | 0 | 0 | 0 | 0 | 1 | 0 | 0 | 0 |
| Marianne3 | 0 | 0 | 0 | 0 | 0 | 0 | 1 | 0 | 0 | 0 |
| Petite Marianne | 0 | 0 | 0 | 0 | 0 | 0 | 1 | 0 | 0 | 0 |
| Madamas | 0 | 0 | 0 | 0 | 0 | 0 | 0 | 0 | 0 | 0 |
| Paria7 | 0 | 0 | 0 | 0 | 0 | 0 | 1 | 0 | 0 | 0 |
| San Souci | 0 | 0 | 0 | 0 | 1 | 0 | 1 | 0 | 0 | 0 |
| Mission | 0 | 0 | 0 | 0 | 1 | 0 | 1 | 0 | 0 | 0 |
| El Cedro 2 | 0 | 0 | 0 | 0 | 0 | 0 | 1 | 0 | 0 | 0 |
| Guanapo Quarry | 1 | 1 | 1 | 1 | 0 | 1 | 1 | 0 | 1 | 1 |
| Lopinot | 1 | 1 | 1 | 1 | 0 | 0 | 1 | 0 | 0 | 0 |
| Lower Arima | 1 | 1 | 1 | 1 | 0 | 1 | 1 | 0 | 1 | 1 |
| Upper Arima | 1 | 1 | 1 | 0 | 0 | 0 | 1 | 0 | 0 | 0 |
| Santa Cruz | 1 | 1 | 1 | 1 | 0 | 0 | 1 | 0 | 0 | 1 |
| Lower Aripo | 1 | 1 | 1 | 1 | 0 | 1 | 1 | 1 | 0 | 1 |
| Upper Naranjo | 0 | 0 | 0 | 0 | 0 | 0 | 1 | 0 | 0 | 0 |
| Blue Basin | 0 | 0 | 0 | 0 | 0 | 0 | 1 | 0 | 0 | 0 |

**Table S8**. Parasites reported infecting Trinidadian guppy (*Poecilia reticulata*) under natural, captive and experimental settings.

|  | **Species** | **Life cycle** | **Report type** | **Location** | **Infection site** | **Source** |
| --- | --- | --- | --- | --- | --- | --- |
| Fungi | |  |  |  |  |  |
|  | *Branchiomyces sanguinis* | D | - | - | - | (Hoffman, 1999) |
|  | Fungi sp. | D | W | Trinidad | Body surface | (Stephenson et al., 2015) |
|  | *Penicillium* sp. | D | C | Iran | Body surface | (Momeni Shahraki et al., 2014) |
|  | *Saprolegnia* sp. | D | C | Iran | Body surface | (Momeni Shahraki et al., 2014) |
|  |  |  |  |  |  |  |
| Protozoa | |  |  |  |  |  |
|  | *Ambiphyra* spp. | D | C | Turkey | Body surface | (Kayis et al., 2005) |
|  | *Apiosoma* spp. | D | W | Trinidad | Body surface | (Stephenson et al., 2015) |
|  | *Chilodonella* sp. | D | W | Trinidad | Fins | (Kennedy et al., 1987) |
|  | *Cryptobia* sp | D | C | Philippines | Gills/body surface | (Arthur and Lumanlan-Mayo, 1997) |
|  | *Cryptocotyle* sp. | D | C | Pakistan | Gills | (Iqbal and Haroon, 2014) |
|  | *Cryptosporidium huwi* | D | C | Australia | Gut | (Ryan et al., 2015) |
|  | *Ichthyobodo necator* | D | C | Turkey | Body surface | (Kayis et al., 2009) |
|  | *Ichthyophthirioides browni* | D |  |  |  | (Roque and Depuytor, 1966) |
|  | *Ichthyophthirius multifiliis* | D | E | - | Body surface | (Sahandi et al., 2013) |
|  | *Ichthyophthirius* spp. | D | W | Trinidad | Body surface | (Stephenson et al., 2015) |
|  | *Myxobolus nuevoleonensis* | D | W | Mexico/Trinidad | Caudal bones, gill arch | (Segovia-Salinas et al., 1991) |
|  | *Myxobolus* sp. | D | W | Trinidad | Spine | Present study |
|  | *Oodinium pillularis* | D | - | - | - | (Hoffman, 1999) |
|  | *Ophryoglena* sp. | D | - | - | Body surface | (Rogers and Gaines, 1975) |
|  | *Tetrahymena corlissi* | D | C | USA | Body surface | (Hoffman et al., 1975) |
|  | *Tetrahymena* sp. | D | C | Singapore | Skin/gills/internal organs | (Pimenta Leibowitz et al., 2005) |
|  | *Trichodina nigra* | D | C | Sri Lanka | Body surface | (Thilakaratne et al., 2003) |
|  | *Trichodina* spp. | D | W | Trinidad | Fins, body surface | (Stephenson et al., 2015) |
|  |  |  |  |  |  |  |
| Monogenea | |  |  |  |  |  |
|  | *Dactylogyrus* cf*. vastator* | D | C | Sri Lanka | Gills | (Thilakaratne et al., 2003) |
|  | *Dactylogyrus extensus* | D | C | Pakistan | Gills | (Iqbal and Haroon, 2014) |
|  | *Gyrodactylus bullatarudis* | D | W | Trinidad | Fins, body surface | (Harris and Lyles, 1992) |
|  | *Gyrodactylus medius* | D | C | England | - | (Hoffman, 1999) |
|  | *Gyrodactylus poeciliae* | D | W | Trinidad | Fins, body surface | (Xavier et al., 2015) |
|  | *Gyrodactylus turnbulli* | D | W | Trinidad | Fins, body surface | (Harris and Lyles, 1992) |
|  | *Salsuginus* sp. | D | W | Mexico | Gills | (Mendez et al., 2010) |
|  | *Urocleidoides reticulatus* | D | W | Trinidad | Gills | Present study |
|  |  |  |  |  |  |  |
| Trematoda | |  |  |  |  |  |
|  | *Ascocotyle paratenuicollis** | I | W | Venezuela | Intestine | (Nasir et al., 1970) |
|  | *Apatemon graciliformis** | I | E | Guadeloupe | Ovary | (Combes and Nassi, 1977) |
|  | *Azygia sebago* | I | E | - | - | (Stunkard, 1956) |
|  | *Bolbophorus confusus** | I | - | - | - | (Hoffman, 1999) |
|  | *Bolbophorus damnificus** | I | E | USA | - | (Levy et al., 2002) |
|  | *Bolbophorus* sp.*** | I | E | USA | - | (Levy et al., 2002) |
|  | *Centrocestus formosanus** | I | W | Mexico | Gills | (Salgado-Maldonado et al., 2005) |
|  | *Cercaria udoi* | I | - | Venezuela | - | (Nasir et al., 1969) |
|  | *Clinostomum complanatum** | I | W | Mexico | Mesentery, muscle | (Mendez et al., 2010) |
|  | *Cryptocotyle* sp.*** | I | C | Pakistan | Gills | (Iqbal and Haroon, 2014) |
|  | *Diplostomum pseudospathaceum** | I | E | - | Lens | (Niewiadomska, 1986) |
|  | *Diplostomum spathaceum** | I | E | - | Lens | (Brassard et al., 1982) |
|  | *Echinochasmus donaldsoni** | I | E | - | - | (Beaver, 1941) |
|  | *Echinochasmus zubedakhaname** | I | W | Venezuela | Mesentery | (Nasir and Diaz, 1968) |
|  | *Euclinostomum* sp.*** | I | C | Thailand | Muscle, liver | (Laoprasert et al., 2006) |
|  | *Guaicaipuria pseudoconcilia** | I | - | Venezuela | - | (Nasir and Dıaz, 1971) |
|  | *Neogogatea pandionis** | I | W | Puerto Rico | Muscle/viscera | (Bunkley-Williams and Williams, 1994) |
|  | *Plagioporus sinitsini* | I | E | - | - | (Hoffman, 1999) |
|  | *Posthodiplostomum minimum** | I | W | Mexico | Mesentery, muscle | (Mendez et al., 2010) |
|  | *Posthodiplostomum nanum** | I | E | Brazil | Body cavity | (López-Hernández et al., 2018) |
|  | *Posthodiplostomum* sp.*** | I | W | Trinidad | Body cavity | Present study |
|  | *Pygidiopsis anterouteria** | I | W | Venezuela | Mesentery | (Nasir and Dıaz, 1971) |
|  | *Ribeiroia* *marina** | I | - | West Indies | - | (Hoffman, 1999) |
|  | *Ribeiroia* *ondatrae** | I | E | - | - | (Hoffman, 1999) |
|  | *Ribeiroia* sp.*** | I | E | - | - | (Pinto et al., 2013) |
|  | *Saccocoelioides tarpazensis* | I | W | Venezuela | Gut | (Díaz and González, 1990) |
|  | *Stephanoprora aylacostoma** | I | E | - | Gills | (Ostrowski de Núñez and Quintana, 2008) |
|  | *Stephanoprora denticulata** | I | E | - | Gills | (Nasir and Scorza, 1968) |
|  | *Stephanoprora paradenticulata** | I | E | - | Gills | (Nasir and Rodriguez, 1969) |
|  | Strigeoid metacercaria*** | I | W | Hawai'i | - | (Font and Tate, 1994) |
|  | *Tylodelphys* sp.*** | I | W | Mexico | Body cavity | (Mendez et al., 2010) |
|  |  |  |  |  |  |  |
| Cestoidea | |  |  |  |  |  |
|  | *Bothriocephalus acheilognathi* | I | W | Hawai'i, Puerto Rico | - | (Font and Tate, 1994) |
|  | *Gryporhynchidae* gen. sp.*** | I | W | Mexico | Gall bladder | (Mendez et al., 2010) |
|  | *Haplobothrium globuliforme* | I | E | - | - | (Hoffman, 1999) |
|  | *Ophiovalipora minuta** | I | C | Puerto Rico | Visceral organs/mesentery | (Bunkley-Williams and Williams, 1994) |
|  | *Valipora* sp.* | I | W | Mexico | Gall bladder | (Mendez et al., 2010) |
|  |  |  |  |  |  |  |
| Nematoda | |  |  |  |  |  |
|  | *Aonchotheca philippinensis* | I | W | Phillipines | Digestive tract mucosa/liver | (Arthur and Lumanlan-Mayo, 1997) |
|  | *Camallanus cotti* | I | C | Brazil | Gut | (Alves et al., 2000) |
|  | *Camallanus* spp. | I | W | Trinidad | Gut | (Stephenson et al., 2015) |
|  | *Capillaria* spp. | I | C | Sri Lanka | Gut | (Thilakaratne et al., 2003) |
|  | *Contracaecum multipapillatum** | I | E | - | Gut | (Huizinga, 1967) |
|  | *Contracaecum spiculigerum** | I | E | - | Gut | (Huizinga, 1966) |
|  | *Eustrongylides ignotus** | I | W | Puerto Rico | Body Cavity/muscle | (Bunkley-Williams and Williams, 1994) |
|  |  |  |  |  |  |  |
| Crustacea | |  |  |  |  |  |
|  | *Argulus foliaceus* | D | C | Iran | Body surface | (Momeni Shahraki et al., 2014) |
|  | *Ergasilus ceylonensis* | D | C | Sri Lanka | Gills | (Thilakaratne et al., 2003) |
|  | *Lernaea cyprinacea* | D | C | Mexico | Body surface | (Vega-Villasante et al., 2011) |
|  | *Lernaea hesaragattensis* | D | C | India | Body surface | (Srinivasachar and Sundarabai, 1971) |
|  |  |  |  |  |  |  |
| Mollusca | |  |  |  |  |  |
|  | *Lasmigona compressa* | I | E | - |  | (Tompa, 1979) |
|  | *Anodonta grandis* | I | W | USA | Gill/Fins | (Bunkley-Williams and Williams, 1994) |

*= larval parasite with maturation in birds.

**References**

Alves, D., Luque, J.L., Paraguassú, A.R., Marques, F.A., 2000. Ocorrência de *Camallanus cotti* (Nematoda: Camallanidae) parasitando o guppy, *Poecilia reticulata* (Osteichthyes: Poeciliidae) no Brasil. Revista da Universidade Rural – Série Ciências da Vida 22, 77–79.

Arthur, J.R., Lumanlan-Mayo, S., 1997. Checklist of the parasites of fishes of the Philippines, FAO fisheries technical paper. Rome.

Beaver, P.C., 1941. The life history of Echinochasmus donaldsoni n. sp., a trematode (Echinostomidae) from the pied-billed grebe. The Journal of Parasitology 27, 347–355.

Brassard, P., Rau, M.E., Curtis, M.A., 1982. Infection dynamics of Diplostomum spathaceum cercariae and parasite-induced mortality of fish hosts. Parasitology 85, 489–493.

Bunkley-Williams, L., Williams, E.H., 1994. Parasites of Puerto Rican freshwater sport fishes. Department of Natural and Environmental Resources, San Juan, PR.

Combes, C., Nassi, H., 1977. Metacercarial dispersion and intracellular parasitism in a strigeid trematode. International journal for parasitology 7, 501–503.

Díaz, M.T., González, T., 1990. Life cycle of Saccocoelioides tarpazensis n. sp.(Trematoda: Haploporidae). Acta Científica Venezolana 41, 327–336.

Font, W.F., Tate, D.C., 1994. Helminth parasites of native Hawaiian freshwater fishes: an example of extreme ecological isolation. The Journal of parasitology 682–688.

Harris, P.D., Lyles, A.M., 1992. Infections of Gyrodactylus bullatarudis and Gyrodactylus turnbulli on guppies (Poecilia reticulata) in Trinidad. The Journal of parasitology 912–914.

Hoffman, G.L., 1999. Parasites of North American freshwater fishes. Cornell University Press, Ithaca, NY.

Hoffman, G.L., Landolt, M., Camper, J.E., Coats, D.W., Stookey, J.L., Burek, J.D., 1975. A disease of freshwater fishes caused by Tetrahymena corlissi Thompson, 1955, and a key for identification of holotrich ciliates of freshwater fishes. The Journal of parasitology 217–223.

Huizinga, H.W., 1967. The life cycle of Contracaecum multipapillatum (von Drasche, 1882) Lucker, 1941 (Nematoda: Heterochelidae). The Journal of parasitology 368–375.

Huizinga, H.W., 1966. Studies on the life cycle and development of Contracaecum spiculigerum (Rudolphi, 1809)(Ascaroidea: Heterocheilidae) from marine piscivorous birds. Journal of the Elisha Mitchell Scientific Society 181–195.

Iqbal, Z., Haroon, F., 2014. Parasitic infections of some freshwater ornamental fishes imported in Pakistan. Pakistan J. Zool 46, 651–656.

Kayis, S., Balta, F., Yandi, I., Akhan, S., 2005. Costia necatrix ve Ambiphyra spp. ile enfeste olmus *Lebistes* (*Poecilia reticulata*) baliklarinda formaldehit uygulamasi. Turkish Journal of Aquatic Life 4, 527–529.

Kayis, S., Ozcelep, T., Capkin, E., Altinok, I., 2009. Protozoan and metazoan parasites of cultured fish in Turkey and their applied treatments. Israeli Journal of Aquaculture - Bamidgeh 61, 93–102.

Kennedy, C.E.J., Endler, J.A., Poynton, S.L., McMinn, H., 1987. Parasite load predicts mate choice in guppies. Behavioral Ecology and Sociobiology 21, 291–295.

Laoprasert, T., Naksuwon, S., Maneepitaksanti, W., Chinabut, S., 2006. Parasitic digenean *Euclinostomum* sp. (Trematoda: Clinostomatidae) in guppy (*Poecilia reticulata*). Warasan Kan Pramong.

Levy, M.G., Flowers, J.R., Poore, M.F., Mullen, J.E., Khoo, L.H., Pote, L.M., Paperna, I., Dzikowski, R., Litaker, R.W., 2002. Morphologic, pathologic, and genetic investigations of *Bolbophorus* species affecting cultured channel catfish in the Mississippi Delta. Journal of Aquatic Animal Health 14, 235–246. https://doi.org/10.1577/1548-8667(2002)014<0235:MPAGIO>2.0.CO;2

López-Hernández, D., Locke, S.A., de Melo, A.L., Leite Rabelo, E., Pinto, H.A., 2018. Molecular, morphological and experimental assessment of the life cycle of *Posthodiplostomum nanum* Dubois 1937 (Trematoda: Diplostomidae) from Brazil, with phylogenetic evidence of paraphyly of the genus *Posthodiplostomum* Dubois, 1936. Infection, Genetics and Evolution In press. https://doi.org/10.1016/j.meegid.2018.05.010

Mendez, O., Salgado-Maldonado, G., Caspeta-Mandujano, J.M., Cabanas-Carranza, G., 2010. Helminth parasites of some freshwater fishes from Baja California Sur, Mexico. Zootaxa 2327, 44–50.

Momeni Shahraki, M., Asgari, M., Khamesipour, F., Raissy, M., 2014. Prevalence of Argulus foliaceus and fungal infections in some ornamental fishes [discus (Symphysodon discus), dwarf gourami (Trichogaster lalius) and guppy (Poecilia reticulata)] in Isfahan City of Iran. Kafkas Üniversitesi Veteriner Fakültesi Dergisi 20.

Nasir, P., De Guevara, D.L., Diaz, M.T., 1970. Study on the trematode larvae of fresh water. XXIV. Partial life-cycle of Ascocotyle paratenuicollis n. sp.(Trematoda: Digenea). Acta Biologica Venezuelica 7, 1–4.

Nasir, P., Diaz, L.M.T., 1968. Studies on freshwater larval trematodes. XVII. The life cycle of Echinochasmus zubedakhaname sp. n. Zeitschrift fur Parasitenkunde 30, 126–133.

Nasir, P., Dıaz, M.T., 1971. Studies on freshwater larval trematodes. XXVII. Partial life cycle of Caiguiria anterouteria gen. n., sp. n., subfam. n.(Trematoda: Digenea). Proceedings of the Helminthological Society of Washington 38.

Nasir, P., Dıaz, M.T., Hamanna, S., 1969. Studies on freshwater larval trematodes. XXV. Two new species of echinostome cercariae. Proceedings of the Helminthological Society of Washington 36, 175–177.

Nasir, P., Rodriguez, L., 1969. Studies on freshwater larval trematodes. XX.The life cycle of *Stephanoprora paradenticulata* n.sp. (Trematoda, Echinostomatidae). Zoologischer Anzeiger 82, 230–244.

Nasir, P., Scorza, J.V., 1968. Studies on freshwater larval trematodes. 18. The life cycle of Stephanoprora denticulata (Rudolphi, 1802) Odhner, 1910 (Trematoda: Digenea: Echinostomatidae). Z Parasitenkd 30, 134–148.

Niewiadomska, K., 1986. Verification of the life-cycles of <i/>Diplostomum spathaceum (Rudolphi, 1819) and *D. pseudospathaceum* Niewiadomska, 1984 (Trematoda, Diplostomidae). Systematic Parasitology 8, 23–31.

Ostrowski de Núñez, M., Quintana, M.G., 2008. The life cycle of Stephanoprora aylacostoma n. sp.(Digenea: Echinostomatidae), parasite of the threatened snail Aylacostoma chloroticum (Prosobranchia, Thiaridae), in Argentina. Parasitology research 102, 647–655.

Pimenta Leibowitz, M., Ariav, R., Zilberg, D., 2005. Environmental and physiological conditions affecting Tetrahymena sp. infection in guppies, Poecilia reticulata Peters. Journal of Fish Diseases 28, 539–547.

Pinto, H.A., Jadin, R.C., Orlofske, S.A., Johnson, P.T.J., Melo, A.L., 2013. Biomphalaria straminea (Mollusca: Planorbidae) as an intermediate host of Ribeiroia sp.(Trematoda: Psilostomidae) in Brazil. The Journal of parasitology 99, 914–918.

Rogers, W.A., Gaines, J.L., 1975. Lesions of protozoan diseases in fish, in: Ribelin, W.E., Migaki, G. (Eds.), The Pathology of Fishes. University of Wisconsin Press, Madison WI, pp. 117–141.

Roque, M., Depuytor, P., 1966. Infraciliature d’un nouvel ophryoglenidae-*Ichthyophthirioides browni*  n.g. n. sp., in: Journal of Protozoology. SOC PROTOZOOLOGISTS 810 E 10TH ST, LAWRENCE, KS 66044, p. 39.

Ryan, U., Paparini, A., Tong, K., Yang, R., Gibson-Kueh, S., O’Hara, A., Lymbery, A., Xiao, L., 2015. Cryptosporidium huwi n. sp.(Apicomplexa: Eimeriidae) from the guppy (Poecilia reticulata). Experimental Parasitology 150, 31–35.

Sahandi, J., Jafaryan, H., Moradi, P., Tadiri, C., 2013. Effect of in-feed probiotic blend on growth performance and infection resistance of the guppy (*Poecilia reticulata*). Bulgarian Journal of Veterinary Medicine 16, 243–250.

Salgado-Maldonado, G., Aguilar-Aguilar, R., Cabanas-Carranza, G., Soto-Galera, E., Mendoza-Palmero, C., 2005. Helminth parasites in freshwater fish from the Papaloapan river basin, Mexico. Parasitology Research 96, 69–89.

Segovia-Salinas, F., Jimenez-Guzman, F., Galaviz-Silva, L., Ramirez-Bom, E., 1991. *Myxobolus nuevoleonensis* n. sp.(Myxozoa: Myxobolidae) parasite of fishes <i>Poecilia mexicana<i/> and <i>P. reticulata<i/> from Rio de la Silla near Monterrey, nuevo Leon, Mexico. Revista Latino-Americana de Microbiologıá 33, 265–269.

Srinivasachar, H.R., Sundarabai, A., 1971. A new copepod parasite <i>Lernaea<i/> sp. nov. on a cyprinodont fish, *Lebistes reticulatus* (Peters). Current Science 40, 453–455.

Stephenson, J.F., Van Oosterhout, C., Mohammed, R.S., Cable, J., 2015. Parasites of Trinidadian guppies: evidence for sex-and age-specific trait-mediated indirect effects of predators. Ecology 96, 489–498.

Stunkard, H.W., 1956. The morphology and life-history of the digenetic trematode, Azygia sebago Ward, 1910. The Biological Bulletin 111, 248–268.

Thilakaratne, I., Rajapaksha, G., Hewakopara, A., Rajapakse, R., Faizal, A.C.M., 2003. Parasitic infections in freshwater ornamental fish in Sri Lanka. Diseases of Aquatic Organisms 54, 157–162.

Tompa, A.S., 1979. Life-cycle completion of the freshwater clam Lasmigona compressa (Bivalvia: Unionidae) in an experimental host, Lebistes reticulatus. Veliger 22, 188–190.

Vega-Villasante, F., Rodríguez-Olivares, K.P., Zuñiga-Medina, L.M., Rojas-Sahagún, C.C., Espinosa-Chaurand, L.D., Rodríguez-Aguilar, J.L., 2011. Infestación por el “piojo ancla” <i>Lernaea cyprinacea<i/> (Crustacea: Copepoda) en el gupi <i>Poecilia reticulata<i/> (Atheriniformes: Poeciliidae): mortalidad y efectos sobre la pigmentación. Avances Sobre Investigaciones Marinas y Acuícolas del Pacifico Tropical Mexicano 2, 161–172.

Xavier, R., Faria, P.J., Paladini, G., Van Oosterhout, C., Johnson, M., Cable, J., 2015. Evidence for cryptic speciation in directly transmitted gyrodactylid parasites of Trinidadian guppies. PloS one 10, e0117096.
